# Rheumatologist’s Views on Oral Stage Dysphagia in adults with Rheumatoid Arthritis of the Temporomandibular Joint: An Irish Perspective

**DOI:** 10.31138/mjr.31.2.225

**Published:** 2020-05-20

**Authors:** Gilheaney Órla, Margaret Walshe

**Affiliations:** Department of Clinical Speech and Language Studies, Trinity College Dublin, Ireland

**Keywords:** Dysphagia, swallowing disorders, deglutition, rheumatology, rheumatoid arthritis, temporomandibular joint, temporomandibular joint disorders

## INTRODUCTION

Temporomandibular disorders (TMDs) affect the temporomandibular joint (TMJ), and may be caused by alterations in the structure and/or function of the TMJ, masticatory muscles, and/or osseous components, causing difficulties such as reduced and painful range of motion, joint sounds, crepitus, or deviation on opening/closing.^1^ Individuals with rheumatoid arthritis (RA) are at risk of developing TMDs,^2^ which can impact on eating, drinking, and swallowing, resulting in oral stage dysphagia (OD).^3^ These TMJ-related difficulties are in addition to other systemic issues that individuals with RA experience, such as burning mouth, taste alterations, or dental health issues, thus increasing the risk of overall eating, drinking and swallowing issues. Recent research suggests that 30.69% and 24.63% of adults with RA affecting the TMJ experience impaired mastication and swallowing, respectively, while masticatory pain (29.97%) and fatigue (21.26%) also affect a sizeable cohort.^4^ However, there is a lack of cohort-specific TMD assessments or evidence-based interventions for the rheumatologist to use with these patients. Research has suggested that as a consequence of this lack of resources, rheumatologists may under-estimate the presence of TMJ involvement, which may result in limited treatment and potential residual deficits.^6–7^ Finally, there is also a lack of clinical guidance to support the rheumatologist in referring patients with non-refractory OD caused by RA-related TMDs to OD specialists.

As a result of these limitations, typical management of OD caused by RA-related TMDs is uncertain, with a potential negative impact on care. However, no previous research has investigated the management of this condition and consequently, and little is known about current care provision and clinicians’ experiences, perspectives and/or satisfaction with this care. The aim of this study was therefore to explore the experience and perspectives of rheumatologists working in Ireland regarding their perceptions of the significance of TMD related OD, typical management and referral practices, and satisfaction with current care provision for this patient group.

## MATERIALS AND METHODS

Ethical approval was granted by the Trinity College Dublin School of Linguistics, Speech and Communication Sciences Research Ethics Committee. A cross-sectional design and online survey were used to explore the perspectives of rheumatologists practising within the Irish healthcare system who were members of the Irish Society for Rheumatology (ISR) during the study period (March – April 2017). Approximately 150 rheumatologists are registered with the ISR. A 13-item online survey was used to seek anonymous information on participant’s demographics, clinical experience, and perceived significance of OD in adults with RA affecting the TMJ. The survey also investigated views about routine evaluation and intervention methods, and multi-disciplinary team (MDT) liaison and referral processes. Finally, participants were invited to provide additional qualitative comments regarding overall perceptions of standards of TMD-related OD care provision (*Table 1*).

**Table 1.** Survey Tool.

**Question number**	**Question**	**Answer Options**
1	What is your profession?	Rheumatologist Medical physician Medical scientist Researcher Other (please specify)
2	In what type of facility do you work?	Large university affiliated teaching hospital Local/General non-teaching hospital Third level academic institution Nursing home Rehabilitation centre Community care Private practice Other setting (Please specify below)
3	How many years of clinical experience do you have?	None <1 year 1–5 years 6–10 years 11–15 years >15 years
4	How significant do you think dysphagia is in TMD patients?	Not at all significant Slightly significant Significant Fairly significant Very significant
5	Do you have clinical experience in the management of patients with dysphagia and TMD? (If no, please proceed to the end of the survey).	Yes No
6	What assessment methods do you routinely use for patients with TMD who report dysphagia?	I do not routinely assess for this Subjective clinical examination Dental examination Self-report questionnaires RDC/TMD protocol Objective imaging assessment Other
7	Do you agree/disagree with these statements? I routinely assess for dysphagia in people with TMD I use a specific assessment protocol with people with TMD and dysphagia This assessment protocol differs based on the presence/absence of dysphagia If dysphagia is suspected in a TMD patient I will refer the patient for videofluoroscopy If dysphagia is suspected in a TMD patient I will refer the patient to appropriate team members	Strongly agree Agree Unsure Disagree Strongly disagree
8	What treatment methods do you routinely use for patients with TMD who report dysphagia?	I do not routinely treat this Compensatory techniques (eg, diet modifications) Non-swallow exercise (eg, chewing exercises) Swallow exercises (eg, effortful swallow) Occlusal appliances Surgery Orthodontics Relaxation techniques Thermal packs Medications Patient education and counselling Other
9	Do you agree/disagree that the following outcomes are important when managing patients experiencing dysphagia and TMDs? Improvement in swallowing Improvement in chewing Improvement in oral intake Improvement in range of motion Reduction of pain Reduction of fatigue Improvement in quality of life Occlusal changes Reduction of parafunctional habits (eg, bruxism) Patient education None of the above	Strongly agree Agree Unsure Disagree Strongly disagree
10	Do you agree/disagree with these statements? I am satisfied with available assessment methods used with TMD patients experiencing dysphagia I am satisfied with available treatment methods used with TMD patients experiencing dysphagia I am satisfied with the overall quality of care typically provided to TMD patients experiencing dysphagia	Strongly agree Agree Unsure Disagree Strongly disagree
11	Which professionals do you typically liaise with when managing patients with dysphagia and RA-related TMDs?	Not applicable Dentist Gastroenterologist Medical physician Rheumatologist Neurologist Surgeon Nurse Dietitian/Clinical nutritionist Speech and language therapist Occupational therapist Physiotherapist Pharmacist Engineer Radiologist Otolaryngologist Medical scientist Other (please specify)
12	Which professionals that you currently do not work with would you like to liaise with when managing these patients?	Dentist Gastroenterologist Medical physician Neurologist Surgeon Nurse Dietitian/Clinical nutritionist Speech and language therapist Occupational therapist Physiotherapist Pharmacist Engineer Radiologist Otolaryngologist Other
13	Do you have any further comments that you would like to share on this topic?	

Descriptive methods were used to analyse findings.

## RESULTS

In total, 16 rheumatologists participated in this survey, with 10 completing it in full, yielding a 62.5% completion rate. While most participants (n=12/75%) reported greater than 15 years of overall clinical experience, only 43.75% (n=7) reported any clinical experience in the management of patients presenting with RA and subsequent TMD-related eating and swallowing problems. A total of 87.5% (n=14) of rheumatologists considered eating and swallowing problems in adults with RA to be a significant clinical consideration in this patient group. However, 66.67% of those who answered this question (n=6) do not typically assess for TMD-related OD, and 50% of those who do assess for these issues were dissatisfied with available assessments. In addition, half of the rheumatologists who completed this question do not typically treat eating and swallowing problems in adults with RA (n=3/50%), with those who do treat these issues relying on a variety of cross-disciplinary interventions, including: compensatory measures such as diet modifications (n=2/33.3%), patient education and counselling (n=1/16.67%), non-swallow exercises (e.g.: chewing activities) (n=1/16.67%), occlusal appliances (n=1/16.67%), and surgery (n=1/16.67%). Half of those who do treat TMD-related OD reported dissatisfaction with available treatment options (n=3; 50%).

No participant reported working in isolation when managing patients with RA and TMD-related OD. Speech and language therapists (SLTs), dietitians, and general dental practitioners were the most common MDT members with whom rheumatologists typically liaised (n=5/83%), with a third of these rheumatologists reporting that they would like to increase their liaison with SLTs and occupational therapists to improve patient clinical outcomes. Two themes emerged during the coding of the qualitative data (*Table 2*). A total of 6 participants completed this section.

**Table 2.** Themes of qualitative data.

**Theme**	**Example**
Perceived low prevalence of eating and swallowing problems in adults with RA-related TMDs	A third (n=2/33.3%) of participants reported that they perceive eating and swallowing problems in adults with RA-related TMDs to occur infrequently (eg, Participant 15: *“[These problems are] actually very rare in modern rheumatology practice*” )
The need for greater MDT support	Half (n=3/50%) of participants emphasised the need for increased MDT interaction and support when managing eating and swallowing problems in adults with RA-related TMDs (eg, Participant 9: “*[We should] refer to SLT and Max Fax surgery* [OMFS] *for advice*”; Participant 12: *“[must] engage with multiple specialists”;* Participant 12: *“[Patients need] correct services” )*

## DISCUSSION

Although a small number of rheumatologists participated, results suggest that OD may be under-identified in this patient group, with low levels of clinical involvement and negative perceptions regarding available resources. Under-identification and low levels of clinical involvement may be due to a range of reasons, including: the perception that TMD-related OD is rare, as highlighted by the additional qualitative comments provided by a third of rheumatologists (eg, *“[TMD-related OD] is actually very rare in modern rheumatology practice”).* However, this view has recently been challenged, with a meta-analysis demonstrating that a range of OD signs and symptoms are commonly reported by participants.^4^ As such, the provision of this new valid and reliable epidemiological evidence may raise clinician awareness of this condition, with subsequent increased levels of identification and perceived significance. In addition to the perceived low significance, it is also suggested that patients may prioritise the reporting of systemic signs and symptoms affecting weight-bearing joints during assessment, rather than TMD-related OD, thus further impacting on identification and treatment rates. This may be due to the severe and overt functional difficulties which patients may experience when weight-bearing joints are affected by RA (eg, mobility difficulties), which may result in only the most severe cases of TMD-related OD being reported. Finally, clinicians may report negative perceptions regarding available clinical tools due to the lack of available resources to support the identification and management of these patients, with potential resultant negative effects on care provision and patient recovery. However, despite these limitations, rheumatologists still consider TMD-related OD to be a significant condition and want to increase their clinical involvement with the wider MDT in order to improve the delivery of care to these patients. This was underlined by a third of participants reporting that “*multiple specialists”* and the “*correct services*” are required for optimal care provision and satisfactory clinical outcomes.

Therefore, this study has underlined that although rheumatologists in Ireland currently report limited clinical involvement and negative perceptions regarding the management of patients with RA who experience TMD-related OD, most consider this to be a clinically significant condition and therefore wish to increase the level of MDT input which these patients typically receive in order to improve their experience of care. Therefore, in order to support this desired change in practice, it is essential that rheumatologists have access to contemporaneous, reliable and valid information regarding the epidemiology and significance of this condition in order to promote awareness, in conjunction with the development of appropriate evidence-based assessment, referral, and management resources, in order to direct and support the provision of future care to patients with TMD-related OD attributed to RA.
